# P2X7 Receptor-Dependent microRNA Expression Profile in the Brain Following Status Epilepticus in Mice

**DOI:** 10.3389/fnmol.2020.00127

**Published:** 2020-08-12

**Authors:** Giorgia Conte, Ngoc T. Nguyen, Mariana Alves, Laura de Diego-Garcia, Aidan Kenny, Annette Nicke, David C. Henshall, Eva M. Jimenez-Mateos, Tobias Engel

**Affiliations:** ^1^Department of Physiology & Medical Physics, Royal College of Surgeons in Ireland (RCSI), Dublin, Ireland; ^2^FutureNeuro, Science Foundation Ireland (SFI) Research Centre for Chronic and Rare Neurological Diseases, Royal College of Surgeons in Ireland (RCSI), Dublin, Ireland; ^3^Walther Straub Institute of Pharmacology and Toxicology, Faculty of Medicine, Ludwig-Maximilians-Universität München, Munich, Germany; ^4^Discipline of Physiology, School of Medicine, Trinity College Ireland, The University of Dublin, Dublin, Ireland

**Keywords:** purinergic signaling, P2X7 receptor, status epilepticus, hippocampus, microRNA

## Abstract

The ionotropic ATP-gated P2X7 receptor is an important contributor to inflammatory signaling cascades *via* the release of Interleukin-1β, as well as having roles in cell death, neuronal plasticity and the release of neurotransmitters. Accordingly, there is interest in targeting the P2X7 receptor for the treatment of epilepsy. However, the signaling pathways downstream of P2X7 receptor activation remain incompletely understood. Notably, recent studies showed that P2X7 receptor expression is controlled, in part, by microRNAs (miRNAs). Here, we explored P2X7 receptor-dependent microRNA expression by comparing microRNA expression profiles of wild-type (wt) and P2X7 receptor knockout mice before and after status epilepticus. Genome-wide microRNA profiling was performed using hippocampi from wt and P2X7 receptor knockout mice following status epilepticus induced by intra-amygdala kainic acid. This revealed that the genetic deletion of the P2X7 receptor results in distinct patterns of microRNA expression. Specifically, we found that in vehicle-injected control mice, the lack of the P2X7 receptor resulted in the up-regulation of 50 microRNAs and down-regulation of 35 microRNAs. Post-status epilepticus, P2X7 receptor deficiency led to the up-regulation of 44 microRNAs while 13 microRNAs were down-regulated. Moreover, there was only limited overlap among identified P2X7 receptor-dependent microRNAs between control conditions and post-status epilepticus, suggesting that the P2X7 receptor regulates the expression of different microRNAs during normal physiology and pathology. Bioinformatic analysis revealed that genes targeted by P2X7 receptor-dependent microRNAs were particularly overrepresented in pathways involved in intracellular signaling, inflammation, and cell death; processes that have been repeatedly associated with P2X7 receptor activation. Moreover, whereas genes involved in signaling pathways and inflammation were common among up- and down-regulated P2X7 receptor-dependent microRNAs during physiological and pathological conditions, genes associated with cell death seemed to be restricted to up-regulated microRNAs during both physiological conditions and post-status epilepticus. Taken together, our results demonstrate that the P2X7 receptor impacts on the expression profile of microRNAs in the brain, thereby possibly contributing to both the maintenance of normal cellular homeostasis and pathological processes.

## Introduction

Purinergic signaling is increasingly recognized to play an important role in diseases of the central nervous system (CNS), including epilepsy (Engel et al., 2016; Burnstock, 2020). Epilepsy, characterized by the occurrence of unprovoked seizures, is one of the most common chronic brain diseases affecting approximately 65 million people worldwide (Moshé et al., 2015). Prolonged seizures (status epilepticus) are harmful to the brain and can trigger lasting changes in brain excitability through cell death, changes in neuronal plasticity, and inflammation (Klein et al., 2018). Although much effort has been invested in identifying what drives these pathological changes, the molecular mechanisms remain incompletely understood.

ATP-gated P2 receptors comprise the ionotropic P2X receptors and metabotropic P2Y receptors. Both classes are increasingly linked to the control of brain excitability in health and disease, including processes that influence seizure generation and the development of epilepsy (Engel et al., 2016; Rassendren and Audinat, 2016; Alves et al., 2018; Burnstock, 2020). Among the P2X receptor family, the P2X7 receptor subtype has attracted the most attention (Henshall and Engel, 2015; Beamer et al., 2017). The P2X7 receptor has the lowest affinity for extracellular ATP suggesting that its activation mainly occurs under pathological conditions where high amounts of ATP are released (Surprenant et al., 1996). The P2X7 receptor is an important driver of inflammation *via* induction of the NLRP3 inflammasome and release of Interleukin-1β (IL-1β) but is also known to affect cellular survival, influence neurotransmitter release and control aberrant synaptic plasticity (Sperlágh et al., 2002; Adinolfi et al., 2005; Di Virgilio et al., 2017; Miras-Portugal et al., 2019). Expression of the P2X7 receptor is found to be elevated in the hippocampus and cortex of rodents subjected to status epilepticus and in the brains of patients with drug-resistant epilepsy (Engel et al., 2012; Jimenez-Pacheco et al., 2013, 2016). While some studies have shown this upregulation to occur primarily on microglia (Rappold et al., 2006; Kaczmarek-Hajek et al., 2018), others have suggested that P2X7 receptor expression is also increased in neurons (Doná et al., 2009; Engel et al., 2012; Jimenez-Pacheco et al., 2016). There is also evidence that P2X7 receptor antagonism can be anticonvulsive and neuroprotective following acute seizures (Engel et al., 2012; Jimenez-Pacheco et al., 2013; Mesuret et al., 2014; Huang et al., 2017; Rodriguez-Alvarez et al., 2017). However, others have observed limited or no protection by P2X7 receptor antagonism (Fischer et al., 2016; Nieoczym et al., 2017), and in some studies P2X7 receptor antagonism was reported to promote seizures (Kim and Kang, 2011; Rozmer et al., 2017). Finally, P2X7 receptor antagonists have also been shown to reduce the duration (Amhaoul et al., 2016) and number (Jimenez-Pacheco et al., 2016) of spontaneous seizures in epileptic rodents. The mechanism(s) of these effects remain, however, poorly understood.

MicroRNAs (miRNAs) are small non-coding RNAs that regulate gene expression at a post-transcriptional level (O’Carroll and Schaefer, 2013). To function, miRNAs are uploaded to the RNA-induced silencing complex (RISC) where Argonaute proteins facilitate complementary base-pairing to target mRNAs resulting in translational repression or degradation of transcripts (Czech and Hannon, 2011). A single miRNA can have numerous targets, either in the same or different pathways. Altered expression of miRNAs has been extensively documented in experimental and human epilepsy (Henshall et al., 2016). Importantly, the targeting of specific miRNAs in animal models has provided compelling evidence that miRNAs influence pathophysiological outcomes after status epilepticus and in chronic epilepsy (Jimenez-Mateos et al., 2012, 2015; Henshall et al., 2016; Tiwari et al., 2018). Notably, the P2X7 receptor was recently identified as a target of miRNAs in the brain (Jimenez-Mateos et al., 2015; Engel et al., 2017; Reigada et al., 2019). How miRNA expression becomes dysregulated following seizures remains, however, incomplete understood.

In the present study, we investigated how genetic deletion of the P2X7 receptor affects miRNA expression in the brain. By using a mouse model of unilateral status epilepticus and P2X7 receptor knockout (*P2rx7^−/−^*) mice, we demonstrate that the loss of the P2X7 receptor alters the expression of several miRNAs under normal physiological conditions and following status epilepticus. Our study demonstrates that P2X7 receptor-controlled downstream signaling pathways include the regulation of an extensive class of miRNAs and thus extends the range of mechanisms by which this receptor influences brain function in health and disease.

## Materials and Methods

### Mouse Models

All animal experiments were performed following the principles of the European Communities Council Directive (2010/63/EU). All procedures carried out in the present manuscript were reviewed and approved by the Research Ethics Committee of the Royal College of Surgeons in Ireland (RCSI; REC 1322) and Health Products Regulatory Authority (HPRA; AE19127/P038; AE19127/P001). Procedures were undertaken as described previously (Torres-Peraza et al., 2013) using 8–12 weeks old male C57Bl/6 wild-type (wt) and *P2rx7^−/−^* mice [6NTac;B6N-P2rx7tm1d(EUCOMM)Wtsi/Ieg] which lack exon 2 of the *P2rx7* gene. Mice were bred at the Biomedical Research Facility (BRF) at RCSI and housed in a controlled facility on a 12-h light/dark cycle at 22 ± 1°C and humidity of 40–60% with food and water provided *ad libitum*. During stereotaxic procedures, mice were anesthetized using isoflurane (5% induction, 1–2% maintenance) and maintained normothermic using a feedback-controlled heat blanket (Harvard Apparatus Limited, Kent, UK). Once fully anesthetized, mice were placed in a stereotaxic frame and a midline scalp incision was performed to expose the skull. A guide cannula (coordinates from Bregma; AP = −0.94 mm, *L* = −2.85 mm) was fixed in place with dental cement. Status epilepticus was induced by microinjection of 0.3 μg KA [in 0.2 μl phosphate-buffered saline (PBS); Sigma-Aldrich, Dublin, Ireland] into the right basolateral amygdala 3.75 mm below the dura. Vehicle-injected control animals received 0.2 μl of PBS. The anticonvulsant lorazepam (6 mg/kg; Wyetch, Taplow, UK) was delivered intraperitoneal (i.p.) 40 min following intra-amygdala KA or vehicle to curtail seizures and to reduce morbidity and mortality. In a subset of mice, which were not included in the miRNA array analysis, the electroencephalogram (EEG) was recorded from cortical implanted electrodes, one on top of each hippocampus with the reference electrode on top of the frontal cortex. EEG was recorded using an Xltek recording system (Optima Medical Limited, Guildford, UK) starting 10 min before the administration of intra-amygdala KA. Hippocampal tissue was obtained from either vehicle-injected control mice or 8 h after intra-amygdala KA injection.

### Electroencephalogram (EEG) Analysis

To analyze seizure onset and EEG frequency and amplitude signal (power spectral density and EEG spectrogram of the EEG data), EEG data were uploaded into Labchart7 software (AD Instruments Limited, Oxford, UK) and analyzed as before (Engel et al., 2018). EEG total power (μV^2^) is a function of EEG amplitude over time and was analyzed by integrating frequency bands from 0 to 100 Hz and the amplitude domain filtered from 0 to 50 mV. The duration of high-frequency (>5 Hz) and high-amplitude (>2 times baseline) polyspike discharges of ≥5 s duration, synonymous with injury-causing electrographic activity (Araki et al., 2002), was counted manually by a reviewer unaware of treatment (Alves et al., 2019).

### RNA Extraction and OpenArray Analysis

Total RNA was extracted from the ipsilateral hippocampus from wt and *P2rx7^−/−^* mice 8 h post-intra-amygdala vehicle or KA using the Trizol method (Engel et al., 2013). RNA quantity was measured using a Nanodrop Spectrophotometer (Thermo Fisher Scientific, Waltham, MA, USA). Only samples with an absorbance ratio at 260/280 between 1.8–2.2 were considered acceptable. RNA degradation was not assessed. RNA dilutions were made up in nuclease-free water.

MiRNA profiling was performed using the OpenArray platform (Thermo Fisher Scientific, Waltham, MA, USA; Jimenez-Mateos et al., 2015). OpenArray reverse transcription reaction was performed according to the manufacturer’s protocol using 1 μg of total RNA from each sample (each sample was a pool of two hippocampi from different mice). Before loading samples onto the OpenArray, cDNA was pre-amplified following the manufacturer’s recommendation. The pre-amplified product was then diluted with 0.1× TE (1/40). Subsequently, 22.5 μl of the diluted pre-amplified product was added to the same volume of 2× Taqman OpenArray Real-time PCR Master Mix (Cat No. 4462164, AB). Finally, the mix of Pre-Amp product and Master-Mix was loaded onto a 384-well OpenArray plate. OpenArray panels were automatically loaded by the OpenArray AccuFill System (Thermo Fisher Scientific, Waltham, MA, USA) and run on a QuantStudio 12 K Flex Real-Time PCR system. 754 murine miRNAs were amplified from each sample together with 16 replicates of four internal controls [ath-miR159a (negative control), RNU48, RNU44, and U6 rRNA]. OpenArray Ct values were normalized to the global mean (GMN). Heat maps were generated using heatmap.2 [RStudio Team (2015). RStudio: Integrated Development for R. RStudio, Inc., Boston, MA, USA^1^] and hierarchical cluster (Cytoscape 3.7.1; Shannon et al., 2003). Only miRNAs were detectable in all samples with a Ct value <28 were used for the analysis (Supplementary Table S1). To detect differences between genotypes and treatment groups two different analyses were performed. To determine differences between all groups (treatment and genotype) data were normalized to the average of the control group (vehicle-injected control wt mice). To identify differences between wt and *P2rx7^−/−^* mice post-status epilepticus, data from *P2rx7^−/−^* mice post-status epilepticus were normalized to data from wt mice post-status epilepticus. Because of our relative low *n* numbers on the OpenArray (*n* = 3 per group), instead of using false discovery rate corrections, miRNAs showing a fold change higher than 1.5 were considered as up-regulated, and miRNAs showing a fold change lower than 0.6 were considered as down-regulated (Supplementary Table S2).

### Individual RT-qPCR (miRNAs)

OpenArray results were validated using small-scale real-time quantitative polymerase chain reaction (RT-qPCR; Mitchell et al., 2008). Extracted total RNA was reverse transcribed using the Reverse Transcription kit (Thermo Fisher Scientific, Waltham, MA, USA) and Taqman primers (mmu-miR-155, ID: 002571; miR-134, ID: 001186) and quantified by RT-qPCR. U6 snRNA (ID: 001973) was selected as an endogenous control. Ct values were normalized to U6 snRNA using the 2^−ΔΔCt^, where ΔΔCt = ΔCt miRNA sample X − ΔCt miRNA reference sample and ΔCt = Ct miRNA (mmu-miR-X) − Ct U6 snRNA.

### Pathway Analysis

For miRNA target identification and Gene Ontology (GO) enrichment analysis, experimentally validated targets were retrieved from miRTarBase Release 7.0 (Chou et al., 2018) and TarBase v.8 (Xiao et al., 2009; Karagkouni et al., 2018) while predicted targets were retrieved from TargetScan Release 7.2 (Agarwal et al., 2015) and miRDB Version 6.0 (Liu and Wang, 2019) and processed as described previously (Raoof et al., 2018) with some modifications. Briefly, prediction scores of TargetScan targets were rescaled between 0 and 1 while those of miRDB targets were rescaled between 0.5 and 1 (since the original miRDB database excluded all targets with scores <50 while the max score is 100). Targets with rescaled prediction scores <0.5 were removed from further analysis. Enrichment analysis of GO terms was only performed on genes that have been targeted by at least two up- or down-regulated miRNAs in each condition (control or status epilepticus) using ReactomePA R/Bioconductor package (Yu and He, 2016). GO terms with adjusted enrichment *p*-values < 0.05 were considered significant.

### Individual RT-qPCR (mRNAs)

Complementary DNA (cDNA) was produced by reverse transcription using SuperScript III reverse transcriptase enzyme (Invitrogen, CA, USA) primed with 50 pmol of random hexamers (Sigma, Dublin, Ireland) using 500 ng of total RNA. qPCR was performed using the QuantiTech SYBR Green kit (Qiagen Limited, Hilden, Germany) and the LightCycler 1.5 (Roche Diagnostics, GmbH, Mannheim, Germany). Each reaction tube contained 2 μl cDNA sample, 10 μl SYBR Green Quantitect Reagent (Qiagen Limited, Hilden, Germany), 1.25 μM primer pair (Sigma, Dublin, Ireland) and RNAse free water (Invitrogen, CA, USA) to a final volume of 20 μl. Using LightCycler 1.5 software, data were analyzed and normalized to the expression of *β-actin*. Primers used (Sigma, Dublin, Ireland): *P2ry1* forward: GTAGGTAGTACGCCAGGGTC, reverse: AAGTAGTTCGGCTGTTCCCA; *c-Fos* forward: GGAATTAACCTGGTGCTGGA, reverse: CATTCAGACCACCTCGACAA; *P2rx2* forward: ATGGGATTCGAATTGACGTT, reverse: GATGGTGGGAATGAGACTGAA; *P2rx4* forward: TATGTGGTCCCAGCTCAGGA, reverse: TCACAGACGCGTTGAATGGA and *β-actin* forward: GGGTGTGATGGTGGGAATGG, reverse: GGTTGGCCTTAGGGTTCAGG.

### Western Blotting

Western blot analysis was performed as described previously (Alves et al., 2017). Lysis buffer (100 mM NaCl, 50 M NaF, 1% Tx-100, 5 mM EDTA pH 8.0, 20 mM HEPES pH 7.4) containing a cocktail of phosphatase and protease inhibitors was used to homogenize hippocampal brain tissue and to extract proteins, which was quantified using a Tecan plate reader at 560 nm. Thirty microgram of protein per sample was separated by sodium dodecyl sulfate-polyacrylamide gel electrophoresis (SDS-PAGE) and immunoblotted using the following primary antibodies: P2X7 receptor (1:200, Cat no: APR-004, Alomone Labs, Jerusalem, Israel), Iba1 (1:400; Cat no: 019-19741; Wako, Neuss, Germany) and β-Actin (1:2,000, Cat no: A5441. Sigma–Aldrich, Dublin, Ireland). Membranes were then incubated with horseradish peroxidase-conjugated secondary antibodies (Jackson ImmunoResearch, Plymouth, PA, USA) and bands visualized using Supersignal West Pico Chemiluminescence Substrate (Pierce, Rockford, IL, USA). Images were captured using a Fuji-Film LAS-3000 (Fuji, Sheffield, UK).

### Statistical Analysis

Statistical analysis was carried out using GraphPad Prism 5.00 and STATVIEW software (5.0.1.0). Data were presented as means ± standard error of the mean (SEM). Analysis of variance (ANOVA) with *post hoc* Fisher’s protected least significant difference test was used to analyze three or more group data. For two-group comparison, Student’s *t*-test was used to determine statistical differences between groups analyzed *via* OpenArray. Significance was accepted at *p < 0.05.

## Results

### Similar Seizure Severity of P2X7 Receptor Knockout Mice and Wild-Type Mice During Status Epilepticus

**Figure 1:**
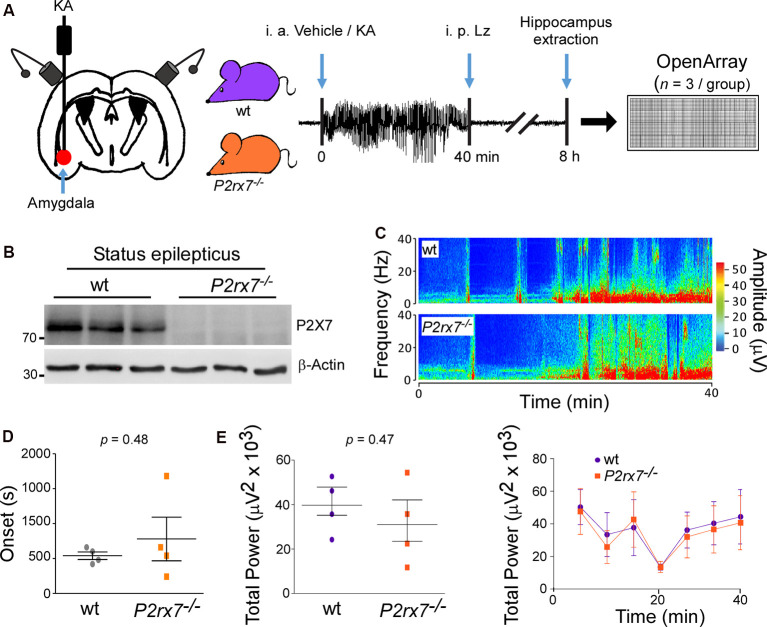
P2X7^−/−^ mice experience similar seizure severity during intra-amygdala Kainic acid when compared to wild-type mice. **(A)** Experimental design: Kainic acid (KA) was injected intra-amygdala to trigger status epilepticus in mice, which was interrupted after 40 min through the injection of the anticonvulsant lorazepam (Lz). Ipsilateral hippocampi were extracted 8 h post status epilepticus from wt and *P2rx7^−/−^* mice, the peak of the P2X7 receptor expression post-status epilepticus in the intra-amygdala KA model. MiRNAs were profiled *via* OpenArray. **(B)** Western blot (*n* = 1 per line) showing the absence of the P2X7 receptor signal (~72 kDa) in the hippocampus of *P2rx7^−/−^* mice 8 h post-status epilepticus. **(C)** Representative electroencephalogram (EEG) recordings presented as heat maps of frequency and amplitude data showing no difference between wt and *P2rx7^−/−^* mice during intra-amygdala KA-induced status epilepticus. **(D)** Graph showing no difference in the onset of seizures between wt mice when compared to *P2rx7^−/−^* mice (*n* = 4 per group). **(E)** Graphs showing no difference in EEG total power between wt and *P2rx7^−/−^* mice during 40 min of status epilepticus starting at the time of intra-amygdala KA injection until the administration of the anticonvulsant lorazepam (*n* = 4 per group).

To establish whether the P2X7 receptor impacts on the miRNA expression profile in the brain, ipsilateral hippocampi from wt and *P2rx7^−/−^* mice were analyzed under physiological conditions (vehicle-injected control mice) and post-status epilepticus using a genome-wide high-throughput qPCR-based miRNA platform (OpenArray; *n* = 3 per group, each sample was a pool of two hippocampi from different mice). The OpenArray is a high-density qPCR assay plate that enables the analysis of hundreds of miRNAs with high reproducibility (Farr et al., 2015). P2X7 receptor-dependent miRNA expression was analyzed 8 h post-status epilepticus, the time-point at which a peak in hippocampal P2X7 receptor expression in the intra-amygdala KA mouse model of status epilepticus has been described (Engel et al., 2012). Status epilepticus was triggered *via* unilateral microinjection of KA into the basolateral amygdala in mice (Mouri et al., 2008; Figure 1A). In this model, status epilepticus leads to neurodegeneration in the brain which is mainly restricted to the ipsilateral brain hemisphere including the cortex and the CA3 subfield of the hippocampus. Hippocampal neurodegeneration is absent in vehicle-injected control mice (Mouri et al., 2008). Western blotting confirmed the absence of the P2X7 receptor expression (~72 kDa) in the ipsilateral hippocampus of *P2rx7^−/−^* mice subjected to status epilepticus (Figure 1B). We first wanted to establish whether the absence of the P2X7 receptor has an impact on the expression of other P2 receptors for which a role during status epilepticus had been suggested previously. This included the P2X family members P2X2 and P2X4 and the P2Y_1_ receptor, which belongs to the P2Y receptor family. Among the P2X receptor family, the P2X4 receptor is the receptor sharing most similarities with the P2X7 receptor (Craigie et al., 2013). Moreover, while expressional changes have been reported for both the P2X2 and P2X4 receptors (Avignone et al., 2008; Engel et al., 2012), P2X4 receptor deficiency has also been shown to aggravate seizure-induced neurodegeneration (Ulmann et al., 2013). In contrast, the P2Y_1_ receptor is involved in both seizure generation and seizure-induced cell death (Simoes et al., 2018; Alves et al., 2019). Interestingly, P2Y_1_ receptor expression has been observed to be particularly increased on microglia post-status epilepticus (Alves et al., 2019), the main cell type expressing the P2X7 receptor (Kaczmarek-Hajek et al., 2018). qPCR revealed that *P2rx7^−/−^* mice showed normal transcript levels of P2X2 and P2X4 receptors in the hippocampus (Supplementary Figure S1A). *P2rx7^−/−^* mice showed also similar transcript levels of the P2Y_1_ receptor. Furthermore, *P2rx7^−/−^* mice displayed a reduction in protein levels of the microglial marker Iba1 in the hippocampus (Supplementary Figure S1B), in line with the known role for P2X7 receptor driving microglia activation (Monif et al., 2009). Finally, hippocampal mRNA levels of the neuronal activity-regulated gene *c-Fos* and baseline EEG recordings were similar between wt and *P2rx7^−/−^* mice suggesting that a loss of the P2X7 receptor does not noticeably alter normal brain function (Supplementary Figures S1C,D).

When subjected to intra-amygdala KA-induced status epilepticus, *P2rx7^−/−^* mice experienced a similar seizure phenotype during the 40 min between injection of KA and administration of lorazepam when compared to wt mice [Total power: wt (39,670 ± 6,227 μV^2^) vs. *P2rx7^−/−^*(31,040 ± 9,174 μV^2^), *p* = 0.4659; Amplitude: wt (411.8 ± 38.70 μV) vs. *P2rx7^−/−^*(412.5 ± 76.45 μV), *p* = 0.9931; high-frequency high amplitude (HFHA) polyspiking: wt (682.5 ± 214.2 s) vs. *P2rx7^−/−^*(613.8 ± 252.9 s), *p* = 0.8425; Figures 1C–E and Supplementary Figures S1E,F]. Reinforcing that *P2rx7^−/−^*and wt mice experience a similar seizure severity during status epilepticus, levels of the neuronal activity-regulated miRNA-134 (Jimenez-Mateos et al., 2012) were comparable between genotypes 8 h post-status epilepticus (Supplementary Figure S1G). Since there were no significant differences in seizure severity during status epilepticus, this indicates that differences in miRNA profiles result directly from changes in signaling rather than being secondary to an effect of altered seizure severity.

### Altered miRNA Expression Profile in P2X7 Receptor Knockout Mice Under Physiological Conditions and Following Status Epilepticus

**Figure 2:**
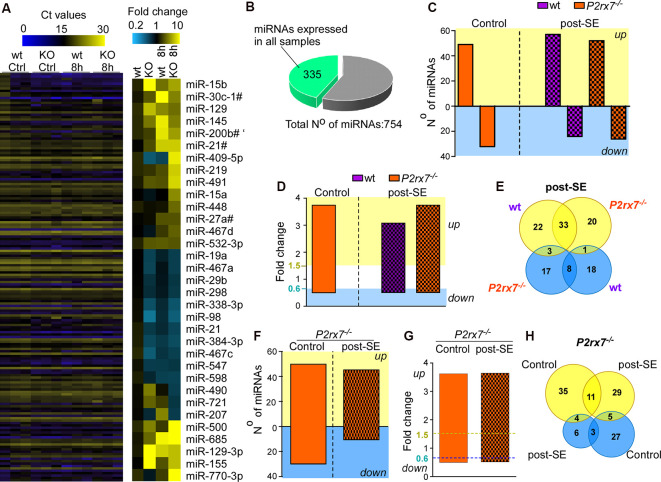
P2X7 receptor-dependent changes in hippocampal miRNA levels. **(A)** Illustrative heat map showing hippocampal miRNA levels in the different groups including vehicle-injected control mice and mice subjected to status epilepticus [wild-type (wt) and *P2rx7^−/−^*(KO)]. Colors on the left indicate Ct values for each miRNA. While blue represents a higher Ct value which corresponds to lower miRNA levels, yellow represents a lower Ct value corresponding to higher miRNA levels. The panel on the right shows miRNAs with the highest fold changes (blue, lower fold-change (FC); yellow, higher FC). Vehicle-injected wt control mice were used as a normalizing control. **(B)** Pie chart indicating the number of miRNAs used for the analysis. The OpenArray analysis included 754 murine miRNAs. Three-hundred and thirty five miRNAs were detected in all hippocampal samples analyzed with Ct values <28. **(C)** Bar chart showing differentially regulated miRNAs in vehicle-injected control mice (*P2rx7^−/−^*) and mice subjected to status epilepticus (wt and *P2rx7^−/−^*). MiRNA expression was normalized to vehicle-injected wt control mice. **(D)** Bar chart showing similar fold changes (FC) of differentially expressed miRNAs among different treatment groups [control and post-status epilepticus (post-SE)] and genotypes (wt and *P2rx7^−/−^*). **(E)** Venn diagram showing the number of up- and down-regulated miRNAs unique and common to wt and *P2rx7^−/−^* mice post-status epilepticus (post-SE). **(F)** Bar chart showing differentially regulated miRNAs in *P2rx7^−/−^* mice [control and post-status epilepticus (post-SE)] when compared to wt mice. **(G)** Bar chart showing similar fold changes (FC) of differentially expressed miRNAs in *P2rx7^−/−^*animals (control and post-status epilepticus). The blue dashed line indicates the cut-off of down-regulated miRNAs, the yellow dashed line indicates the cut-off of up-regulated miRNAs. **(H)** Venn diagram showing the number of up-regulated and down-regulated miRNAs in *P2rx7^−/−^* mice during control conditions and post-status epilepticus (post-SE).

Next, we profiled miRNA expression within the hippocampus of wt and *P2rx7^−/−^* mice (Figure 2A). MiRNAs were included if detected in all samples of all four groups (vehicle-and KA-injected wt and *P2rx7^−/−^* mice). This resulted in 335 miRNAs (Figures 2A,B, and Supplementary Table S1). Demonstrating OpenArray results to reproduce earlier findings, our analysis identified several miRNAs shown to alter their expression following seizures and/or during epilepsy in previous studies (e.g., miR-134, miR-21, and miR-27a* were found to be up-regulated and miR-18a down-regulated post-status epilepticus; Roncon et al., 2015; Cava et al., 2018; Supplementary Figures S1G, S2). We then compared miRNA profiles between vehicle-injected control wt and *P2rx7^−/−^* mice. This revealed that 50 miRNAs are up-regulated and 35 miRNAs are down-regulated in *P2rx7^−/−^* mice (Figure 2C). Then, we investigated to what extent the lack of the P2X7 receptor had an effect on the miRNA expression profile after status epilepticus. When both genotypes subjected to intra-amygdala KA were compared to vehicle-injected control wt mice, 58 miRNAs were up-regulated and 26 miRNAs were down-regulated in wt mice after status epilepticus and 53 miRNAs were up-regulated and 28 miRNAs down-regulated in *P2rx7^−/−^* mice after status epilepticus (Figure 2C). Average fold changes in miRNA expression were similar between conditions and genotypes (Figure 2D and Supplementary Figure S3). Further analysis revealed 33 miRNAs were commonly regulated after status epilepticus between genotypes whereas 22 miRNAs were unique to wt and 20 miRNAs to *P2rx7^−/−^* mice among the up-regulated miRNAs post-status epilepticus. Among the down-regulated miRNA pool, eight were common between both genotypes, 17 miRNAs were unique to *P2rx7^−/−^*and 18 unique to wt mice. Only three miRNAs were up-regulated in wt and down-regulated in *P2rx7^−/−^* mice and one single miRNA was at the same time up- in *P2rx7^−/−^* mice and down-regulated in wt mice (Figure 2E). Interestingly, miRNA expression differences between wt and *P2rx7^−/−^* mice are more pronounced during physiological conditions when compared to post-status epilepticus (85 in control conditions and 59 post-status epilepticus) with more miRNAs being up-regulated under both conditions 50 up- (e.g., miR-671-3p, fold change (FC) = 6.66; miR-129, FC = 4.69) and 35 down-regulated (e.g., miR-431*, FC = 0.27; miR-20b, FC = 0.28) in control conditions and 44 up- (e.g., miR-770-3p, FC = 34.1; miR-409-5p, FC = 14.92) and 13 down-regulated (e.g., miR-721, FC = 0.41; miR-490, FC = 0.46) post-status epilepticus; Figure 2F and Supplementary Table S2). Again, fold changes in miRNA expression were similar between conditions (Figure 2G). Notably, minimal overlap in altered miRNAs was observed between vehicle-injected control *P2rx7^−/−^* mice when compared to *P2rx7^−/−^* mice subjected to status epilepticus with only 11 miRNAs out of 64 common among up-regulated miRNAs and 3 out of 37 among down-regulated miRNAs (Figure 2H). This suggests P2X7 receptor-driven changes in the miRNA profile are dependent on physiological context.

In summary, P2X7 receptor deficiency leads to a distinct miRNA signature in the hippocampus during normal physiology and following status epilepticus with increased miRNA expression being the predominant response under both conditions.

### Pathways Targeted *via* P2X7 Receptor-Dependent miRNAs

We then explored what genes and pathways are potentially regulated *via* P2X7 receptor-dependent miRNAs during physiological conditions and following status epilepticus. To ensure meaningful results, only experimentally validated and reliably predicted target genes (with comparable prediction scores across databases) were taken into account. Our analysis predicted 9,927 genes to be targeted *via* up-regulated miRNAs and 6,144 genes targeted *via* down-regulated miRNAs during physiological conditions. Post-status epilepticus, 8,254 genes were identified to be targeted *via* up-regulated miRNAs and only 235 genes were predicted to be targeted *via* down-regulated miRNAs (Supplementary Table S3).

**Figure 3:**
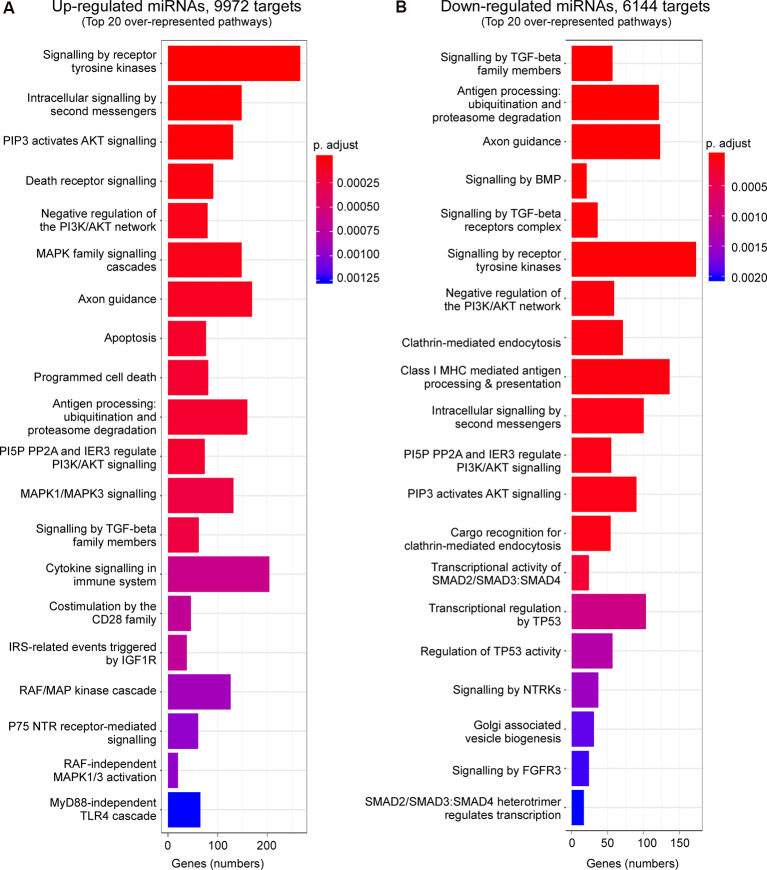
Predicted pathways targeted *via* P2X7 receptor-dependent miRNAs in the hippocampus during control conditions. Bar charts showing the top 20 pathways predicted to be targeted *via* P2X7 receptor-dependent **(A)** up-regulated and **(B)** down-regulated miRNAs in the hippocampus of vehicle-injected control *P2rx7^−/−^* mice.

**Figure 4:**
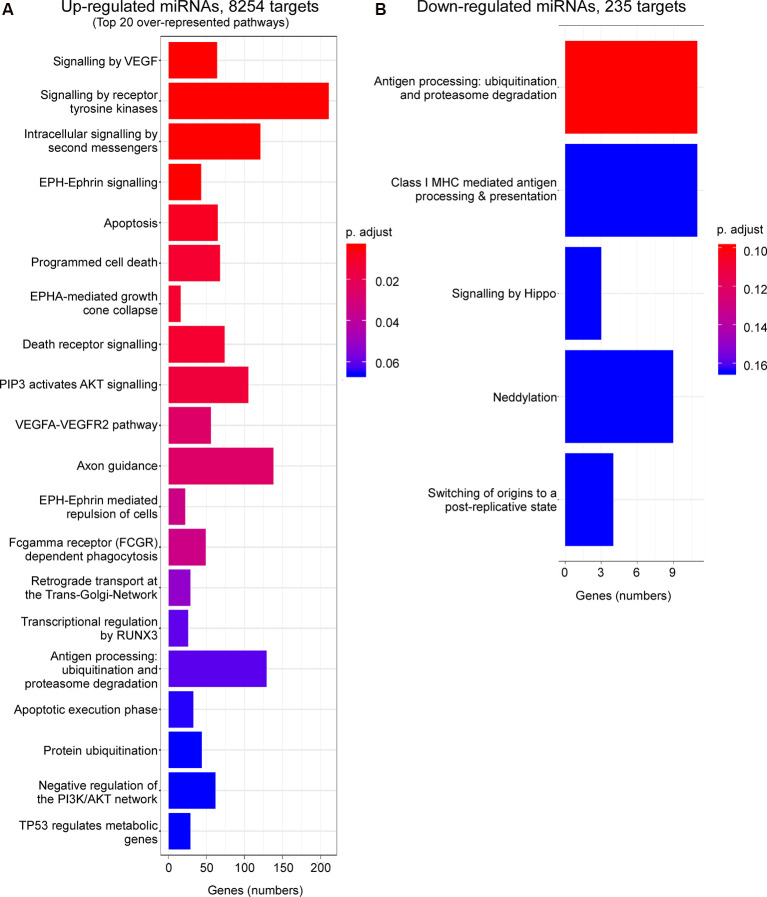
Predicted pathways targeted *via* P2X7 receptor-dependent miRNAs in the hippocampus post-status epilepticus. Bar charts showing pathways predicted to be targeted *via* P2X7 receptor-dependent **(A)** up-regulated and **(B)** down-regulated miRNAs in the ipsilateral hippocampus of *P2rx7^−/−^* mice post-status epilepticus.

Enrichment analysis of GO terms of putative target genes of P2X7 receptor-dependent miRNAs shows that genes involved in signaling pathways (e.g., “Signaling by VEGF,” “Signaling by Receptor Tyrosine Kinases,” “Intracellular signaling by second messengers,” “MAPK family signaling cascades,” “MAPK1/MAPK3 signaling,” “PIP3 activates AKT signaling,” “Signaling by TGF-beta family members”) were overrepresented under both vehicle-injected control conditions (up- and down-regulated miRNAs) and post-status epilepticus (up-regulated miRNAs) with signaling cascades associated with the serine/threonine-specific protein kinase AKT [also known as Protein kinase B (PKB)] particularly abundant. Of note, pathways associated with the mitogen-activated protein kinase (MAPK) were only present among the up-regulated miRNAs in vehicle-injected control mice. Genes involved in the regulation of the immune system (e.g., “Antigen processing: Ubiquitination and Proteasome degradation,” “Class I MHC mediated antigen processing and presentation,” “Cytokine Signalling in Immune system”) are overrepresented in P2X7 receptor-dependent up- and down-regulated miRNAs in vehicle-injected control mice and post-status epilepticus, in line with the P2X7 receptor being a major contributor to inflammatory signaling cascades (Di Virgilio et al., 2017). In contrast, pathways regulating cell death seem to be mainly restricted to the up-regulated miRNA pool (control conditions and post-status epilepticus; e.g., “Apoptosis,” “Programmed cell death,” “Death receptor signaling”; Figures 3, 4 and Supplementary Figures S4, S5). Other pathways include: (a) “Axon guidance” (targeted by both up- and down-regulated miRNAs in control conditions and up-regulated miRNAs after status epilepticus); (b) “EPH-Ephrin signaling,” involved in neuronal migration and targeted by up-regulated miRNAs post-status epilepticus); (c) “Clathrin-mediated endocytosis” and “Regulation of TP53 activity” (targeted by down-regulated miRNAs in control conditions); (d) Phagocytosis “Fcgamma receptor (FCGR) dependent phagocytosis” and “Protein ubiquitination” (targeted by up-regulated miRNAs post-status epilepticus); and (e) “Neddylation” and “Signaling in Hippo” (targeted by down-regulated miRNAs post-status epilepticus; Figures 3, 4 and Supplementary Figures S4, S5). Thus, P2X7 receptor-regulated miRNAs seem to impact on numerous cellular pathways during both physiological and pathological conditions with a strong overrepresentation of genes involved in intracellular signaling pathways, cellular survival, and inflammation processes repeatedly linked to the P2X7 receptor (Kopp et al., 2019).

**Figure 5:**
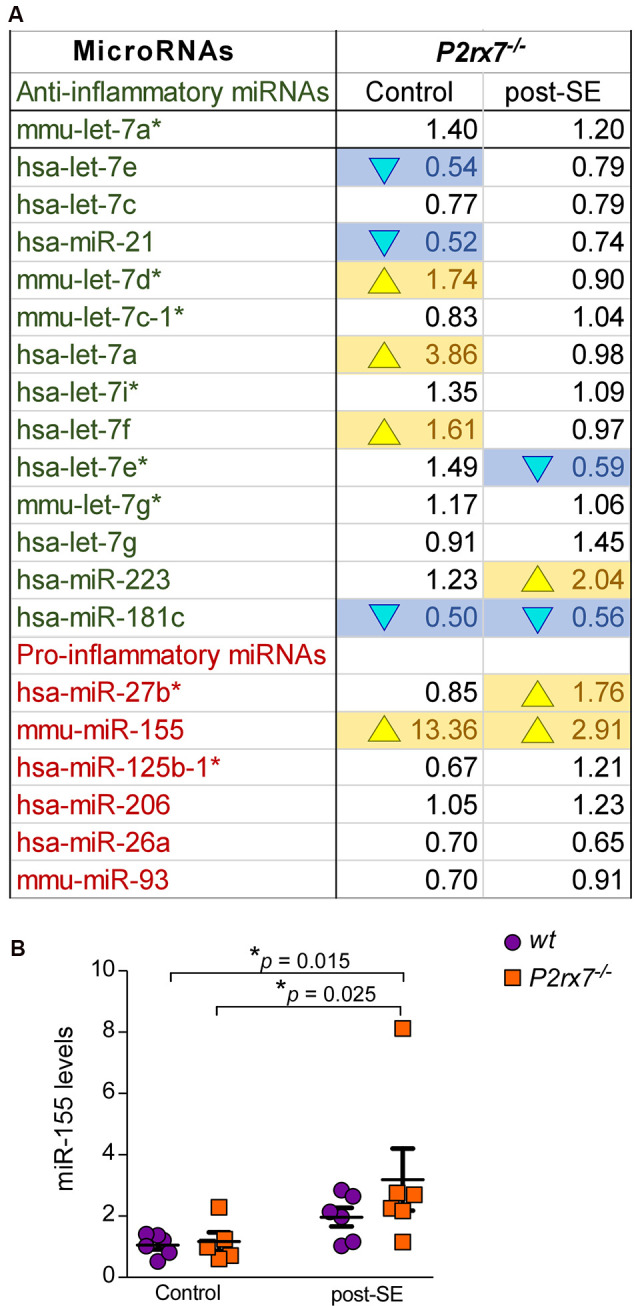
Inflammatory miRNAs regulated *via* the P2X7 receptor.** (A)** Table showing the average fold changes (FC) of anti-inflammatory and pro-inflammatory miRNAs in the ipsilateral hippocampus of *P2rx7^−/−^* mice during control conditions (Control) and post-status epilepticus (post-SE). **(B)** Graph showing hippocampal miR-155 levels in the different treatment groups (*n* = 6 per group; analysis of variance (ANOVA) with Fisher’s *post hoc* test: 1.057 ± 0.1418 (Control, wt) vs. 3.191 ± 1.013 (post-SE, *P2rx7^−/−^*), *F* = 2.987, *df* = 3, *p* = 0.015; 1.167 ± 0.3006 (Control, *P2rx7^−/−^*) vs. 3.191 ± 1.013 (post-SE, *P2rx7^−/−^*), *F* = 2.987, *df* = 3, *p* = 0.025).

Finally, the P2X7 receptor has been described as an important contributor to inflammatory processes (Adinolfi et al., 2018). We, therefore, analyzed specifically expression changes of P2X7 receptor-dependent miRNAs with a known role during inflammation (Figure 5A). While 8 out of 14 anti-inflammatory miRNAs were either up- or down-regulated in *P2rx7^−/−^* mice, only two out of six pro-inflammatory miRNAs showed altered expression in *P2rx7^−/−^* mice (Figure 5A). In line with more miRNAs undergoing expression changes in vehicle-injected control *P2rx7^−/−^* mice, more inflammation-associated miRNAs seem to be dysregulated during physiological conditions when compared to post-status epilepticus (Figure 5A). Inflammatory miRNAs differentially expressed in vehicle-injected *P2rx7^−/−^* mice include: (a) down-regulated anti-inflammatory miRNAs let-7e, miR-21, and miR-181c; (b) up-regulated anti-inflammatory miRNAs let-7d*, let-7a, let-7f; and (c) up-regulated pro-inflammatory miRNA-155. Inflammatory miRNAs differentially expressed in *P2rx7^−/−^* mice post-status epilepticus include: (a) down-regulated anti-inflammatory miRNAs let-7e* and miR-181c; (b) up-regulated anti-inflammatory miRNA miR-223; and (c) up-regulated pro-inflammatory miRNAs miR-27b* and miRNA-155 (Barnett et al., 2016; Gaudet et al., 2018; Gui et al., 2018; Song et al., 2019; Sun et al., 2019; Zhang J. et al., 2019). Among these, miR-155 was detected as one of the most consistently dysregulated miRNA in *P2rx7^−/−^* mice *via* our OpenArray analysis showing increased levels in vehicle-injected *P2rx7^−/−^* control mice and *P2rx7^−/−^* mice subjected to status epilepticus (Figure 5A). Moreover, miR-155 has a well-established role during inflammation (Mahesh and Biswas, 2019) and has repeatedly been associated with epilepsy (Huang et al., 2018; Fu et al., 2019; Zhang W. et al., 2019). While no expression changes could be observed during control conditions, individual qPCR confirmed miR-155 to be upregulated in *P2rx7^−/−^* mice following status epilepticus (Figure 5B). Therefore, in line with the P2X7 receptor being an important contributor to inflammatory processes, miRNAs previously associated with inflammation undergo widespread expression changes in *P2rx7^−/−^* mice.

## Discussion

In the present study, we show that the ATP-gated P2X7 receptor impacts on the miRNA expression profile in the hippocampus during normal physiology and following status epilepticus. While the lack of the P2X7 receptor leads to a predominant up-regulation of miRNAs during physiological and pathological conditions, we found very little overlap in identified miRNAs between both conditions. Our results, therefore, suggest a context-specific contribution of the P2X7 receptor to gene expression in the brain *via* the regulation of miRNAs during both normal physiology and pathology.

Several studies have demonstrated that P2X7 receptor expression in the brain is partly regulated *via* miRNAs including miR-22 and miR-135a (Jimenez-Mateos et al., 2015; Reigada et al., 2019). In line with the P2X7 receptor driving seizures and epilepsy development, suppression of miR-22 in the intra-amygdala KA mouse model led to increased P2X7 receptor expression, an increase in inflammation, and the formation of a secondary epileptic focus (Jimenez-Mateos et al., 2015). Blocking of miR-135a on the other hand protects against excitotoxicity in a model of traumatic spinal cord injury (Reigada et al., 2019). Whether the P2X7 receptor impacts on the miRNA expression profile in the brain has, however, not been investigated to date.

A first unexpected finding of our study was that the absence of the P2X7 receptor leads to a higher number of dysregulated miRNAs under physiological conditions than post-status epilepticus. This is even more remarkable, as previous studies using *P2rx7^−/−^* mice have provided little evidence of altered P2X7 receptor down-stream signaling during physiological conditions with the regulation of cytokines representing one of the few studies reported (Solle et al., 2001; He et al., 2017). Moreover, the affinity of the P2X7 receptor to extracellular ATP is much lower (activation threshold: 0.3–0.5 mM) than that of other P2X receptor subtypes, and extracellular ATP concentrations are only thought to activate the receptor during pathological conditions (Idzko et al., 2014). It is, however, important to keep in mind that our studies used a constitutive *P2rx^−/−^* mouse; therefore, molecular changes may have developed over a prolonged period leading to more pronounced changes than during the 8 h following status epilepticus. An alternative explanation is a possible ceiling effect of dysregulated miRNAs post-status epilepticus masking possible differences between wt and *P2rx7^−/−^* mice.

Another surprising finding is the almost complete lack of overlap in identified P2X7 receptor-regulated miRNAs between physiological and pathological conditions. Again, the reason for this remains elusive. The P2X7 receptor, however, may activate different pathways depending on its activation status and the availability of extracellular ATP, which may increase during status epilepticus (Beamer et al., 2019). The P2X7 receptor may also be expressed in different cell populations according to physiological context, thereby differently affecting their miRNA expression profiles. While the P2X7 receptor has been described to be mainly expressed on microglia and oligodendrocytes during physiological conditions (Collo et al., 1997; Matute et al., 2007; Kaczmarek-Hajek et al., 2018), the P2X7 receptor has also been found on neurons following status epilepticus and during epilepsy (Doná et al., 2009; Engel et al., 2012; Jimenez-Pacheco et al., 2016). To establish the exact cell-specific P2X7 receptor-dependent miRNA expression profile will, however, require the use of cell-specific *P2rx7^−/−^* mice.

What are the molecular mechanisms by which the P2X7 receptor impacts on the expression profile of miRNAs? The P2X7 receptor has been shown to regulate the activity of a variety of different transcription factors [e.g., Nuclear Factor-κB (Nf-κB), Activator protein 1 (AP-1), Glycogen synthase kinase-3β (Barth et al., 2016; Kopp et al., 2019)] and signaling pathways [e.g., MAPK/ERK pathway (Chen et al., 2013)] which may influence the expression of miRNAs. MiRNAs are transcribed as long primary-miRNAs (pri-miRNAs) in the nucleus. Once transported into the cytoplasm, miRNAs are further processed *via* an enzyme called DICER into their mature form. This mature form is then incorporated into the RISC complex for complementary base-pairing with their corresponding target mRNAs (Czech and Hannon, 2011). The activity of DICER is regulated *via* cleavage by proteases, including the calcium-activated protease calpain, and by caspases (Lugli et al., 2005) which are both shown to be activated by the P2X7 receptor (Kong et al., 2005; Jimenez-Mateos et al., 2019). Interestingly, patients with temporal lobe epilepsy presented, despite showing no changes in pri-miRNA levels, reduced levels of mature miRNAs and full-length DICER (McKiernan et al., 2012). Thus, we cannot exclude that the observed P2X7 receptor-dependent alterations of the miRNA expression profiles are due to changes in the processing of pri-miRNAs rather than increased transcription rates of miRNAs. P2X7 receptor-dependent miRNA expression may also be altered *via* other P2X7 receptor-regulated processes including inflammation or, in the case of changes occurring during status epilepticus, differences in the severity of seizures and seizure-induced neurodegeneration between genotypes. However, the latter is unlikely since *P2rx7^−/−^* mice and their wt littermates showed a similar seizure phenotype during status epilepticus. This is an unexpected finding, as a previous study has shown that P2X7 receptor deficiency leads to seizure suppression during status epilepticus in the intra-amygdala KA mouse model (Engel et al., 2012; Jimenez-Pacheco et al., 2013). However, a different *P2rx7^−/−^*mouse model which expresses a P2X7 receptor splice variant in the CNS (Masin et al., 2012) had been used in the previous study. Whether P2X7 receptor-mediated alterations in miRNA levels result in altered functions of miRNAs has not been analyzed. MiRNA expression has however been shown to correlate with miRNA binding to Ago-2 which is the main component of the RISC complex (Martinez and Gregory, 2013). Finally, our results show that the absence of the P2X7 receptor leads to more miRNAs being up-regulated than down-regulated under both physiological and pathological conditions. Again, we do not know the reason behind this. P2X7 receptor deficiency-mediated up-regulation of miRNAs, however, suggests P2X7 receptor deficiency having an overall negative impact on protein expression possibly promoting thereby a protective phenotype (Jimenez-Mateos and Henshall, 2009). Another possibility is that the loss of the *P2rx7* gene “de-represses” miRNAs normally bound to the *P2rx7* mRNA, thereby increasing their abundance.

The P2X7 receptor has been involved in numerous molecular processes regulating vital cellular functions with a particular emphasis on pathological conditions, which in part can probably be attributed to its low affinity to extracellular ATP (Surprenant et al., 1996; Jimenez-Mateos et al., 2019). The P2X7 receptor has been described as a gatekeeper of inflammation regulating the activation of the NLRP3 inflammasome and the release of various cytokines (Di Virgilio et al., 2017). In addition to inflammation, the P2X7 receptor has, however, also been implicated in numerous other pathological processes pertinent to epileptogenesis including disruption of the blood-brain barrier, neurogenesis, regulation of neurotransmitter release and cellular survival (Sperlágh and Illes, 2014; Barros-Barbosa et al., 2016; Miras-Portugal et al., 2019). Most notably, pathway analysis of genes targeted *via* P2X7 receptor-dependent miRNAs revealed that most identified pathways have previously been associated with the P2X7 receptor including intracellular signaling (e.g., VEGF or AKT), inflammation and cell death (Amoroso et al., 2015; Di Virgilio et al., 2017; Miras-Portugal et al., 2019). Why genes involved in signaling pathways are strongly overrepresented among up-regulated miRNA targets and are not present among down-regulated miRNAs in *P2rx7^−/−^* mice post-status epilepticus remains to be determined; however, decreasing the activation of these pathways may serve a neuroprotective strategy reducing cellular energy depletion (Jimenez-Mateos and Henshall, 2009). Interestingly, inflammatory pathways are particularly enriched among P2X7 receptor-dependent miRNA targets, suggesting altered immune responses in *P2rx7^−/−^* mice, which is in line with previous studies showing reduced cytokine release in these mice (Solle et al., 2001). Regulation of the inflammatory process by the P2X7 receptor is further supported by the fact that P2X7 receptor deficiency leads to altered levels of pro- and anti-inflammatory miRNAs. Evidence for the P2X7 receptor regulating inflammatory signaling during seizures and epilepsy stems from data showing that blocking of the P2X7 receptor during status epilepticus leads to a decrease in the release of the proconvulsant cytokines IL-1β and Tumor necrosis factor-α (TNF-α) and a reduction in Nf-κB-mediated inflammation in the hippocampus (Kim et al., 2011; Engel et al., 2012; Huang et al., 2017). Moreover, P2X7 receptor antagonist-treated epileptic mice show a strong decrease in both astrogliosis and microgliosis (Jimenez-Pacheco et al., 2016). In contrast, genes involved in apoptotic pathways are predominantly overrepresented among up-regulated miRNAs, suggesting that *P2rx7^−/−^* mice could be protected from cell death, which is in good agreement with a pro-apoptotic function of the P2X7 receptor and the observed neuroprotection *via* P2X7 receptor antagonism from seizure-induced cell death (Adinolfi et al., 2005; Engel et al., 2012; Miras-Portugal et al., 2019). Other pathways linked to the P2X7 receptor include a role in “Axon guidance” in line with previous studies showing that blocking of the P2X7 receptor promotes axon growth and axon branching (Díaz-Hernandez et al., 2008). Of note, genes of the “Signaling by Hippo” pathway are overrepresented within the down-regulated miRNA pool post-status epilepticus. This is in line with a recent study showing the P2X7 receptor driving proliferation following seizures (Rozmer et al., 2017). Importantly, processes predicted to be targeted *via* P2X7 receptor-regulated miRNAs have all previously been associated with status epilepticus. This includes particularly pathways involved in inflammation (Vezzani et al., 2019), but also pathways linked to intracellular signalings such as the MAPK pathway (Hansen et al., 2014), cell death (Engel et al., 2010) and aberrant neurogenesis (Cho et al., 2015). Interestingly, the P2X7 receptor itself has been shown to regulate neurogenic processes following status epilepticus (Rozmer et al., 2017) as has also miR-22 (Beamer et al., 2018), which targets the P2X7 receptor during status epilepticus (Engel et al., 2017). Thus, the P2X7 receptor may impact on the epileptic phenotype directly and indirectly *via* the activation or suppression of selected miRNAs.

Possible limitations of our study include the fact that we have not established whether P2X7 receptor-regulated miRNAs contribute to pathological changes following status epilepticus previously attributed to the P2X7 receptor. Because P2X7 receptor-dependent miRNAs seem to impact mainly on pathways/networks linked to the P2X7 receptor, it is, however, tempting to speculate that these miRNAs act as an additional mechanism to fine-tune and/or amplify P2X7 receptor signaling. It is also important to keep in mind that our approach used a constitutive knockout of the P2X7 receptor. While *P2X7^−/−^* mice showed similar brain levels of different P2 receptors in the brain, we cannot exclude that the observed changes in miRNA expression are in part due to either developmental alterations or possible compensation mechanisms. Thus, our results should be validated in future studies using either conditional P2X7 receptor knockout mice or P2X7 receptor antagonists. While we acknowledge the low n number for our OpenArray analysis, our study aimed to provide the proof-of-principle data that P2X7 receptor signaling impacts on the miRNA expression profile in the brain extending thereby the potential mechanisms by which the P2X7 receptor impacts on brain function in health and during pathological processes. Also, our analysis has focused on the hippocampus which is one of the main brain structures affected within the intra-amygdala mouse model and patients with temporal lobe epilepsy (Chang and Lowenstein, 2003; Mouri et al., 2008). However, seizures may also affect extra-hippocampal brain areas, such as the cortex, which also show increased expression of the P2X7 receptor (Jimenez-Pacheco et al., 2013). Lastly, rather than decreasing, P2X7 receptor expression levels increase during pathology (Beamer et al., 2017; Miras-Portugal et al., 2019). While outside of the scope of the present manuscript, the recent development of the P2X7 receptor overexpressing mice (Kaczmarek-Hajek et al., 2018) may allow the analysis of the effects of increased P2X7 receptor expression/function on miRNA profiles in future studies.

In summary, our data demonstrate that P2X7 receptor signaling affects the expression profile of miRNAs in the brain thereby possibly contributing to the gene expression landscape during normal physiology and pathology which should be taken into consideration when analyzing P2X7 receptor-driven molecular pathomechanisms.

## Data Availability Statement

The original contributions presented in the study are publicly available. This data can be found in the Gene Expression Omnibus: https://www.ncbi.nlm.nih.gov/geo/query/acc.cgi?acc=GSE153204, accession number GSE153204.

## Ethics Statement

The animal study was reviewed and approved by Research Ethics Committee of the Royal College of Surgeons in Ireland.

## Author Contributions

GC carried out qPCRs and Western blotting, analyzed the OpenArray, and wrote parts of the manuscript. NN carried out pathway analysis. LD-G carried out Western blotting. MA analyzed EEG, performed qPCR and carried out Western blotting. AK generated heat maps. AN bred and provided P2X7 knockout mice and edited the manuscript. DH edited manuscript. EJ-M performed experiments with OpenArray and edited manuscript. TE supervised the study, carried out *in vivo* work and wrote the manuscript.

## Conflict of Interest

The authors declare that the research was conducted in the absence of any commercial or financial relationships that could be construed as a potential conflict of interest.

## Footnotes

^1^http://www.rstudio.com/
