# Supplementary material for: P2X7 Receptor-Dependent microRNA Expression Profile in the Brain Following Status Epilepticus in Mice
Source: Front Mol Neurosci. 2020 Aug 12;13:127. doi: 10.3389/fnmol.2020.00127 (PMC7485385; doi:10.3389/fnmol.2020.00127)
Supplement: Supplementary file 1 [file Presentation_1.pdf]

Supplementary File

Supplementary Figure S1. *Similar seizure phenotype between P2rx7$^{-/-}$ mice and wild-type mice during intraamygdala KA-induced status epilepticus.*

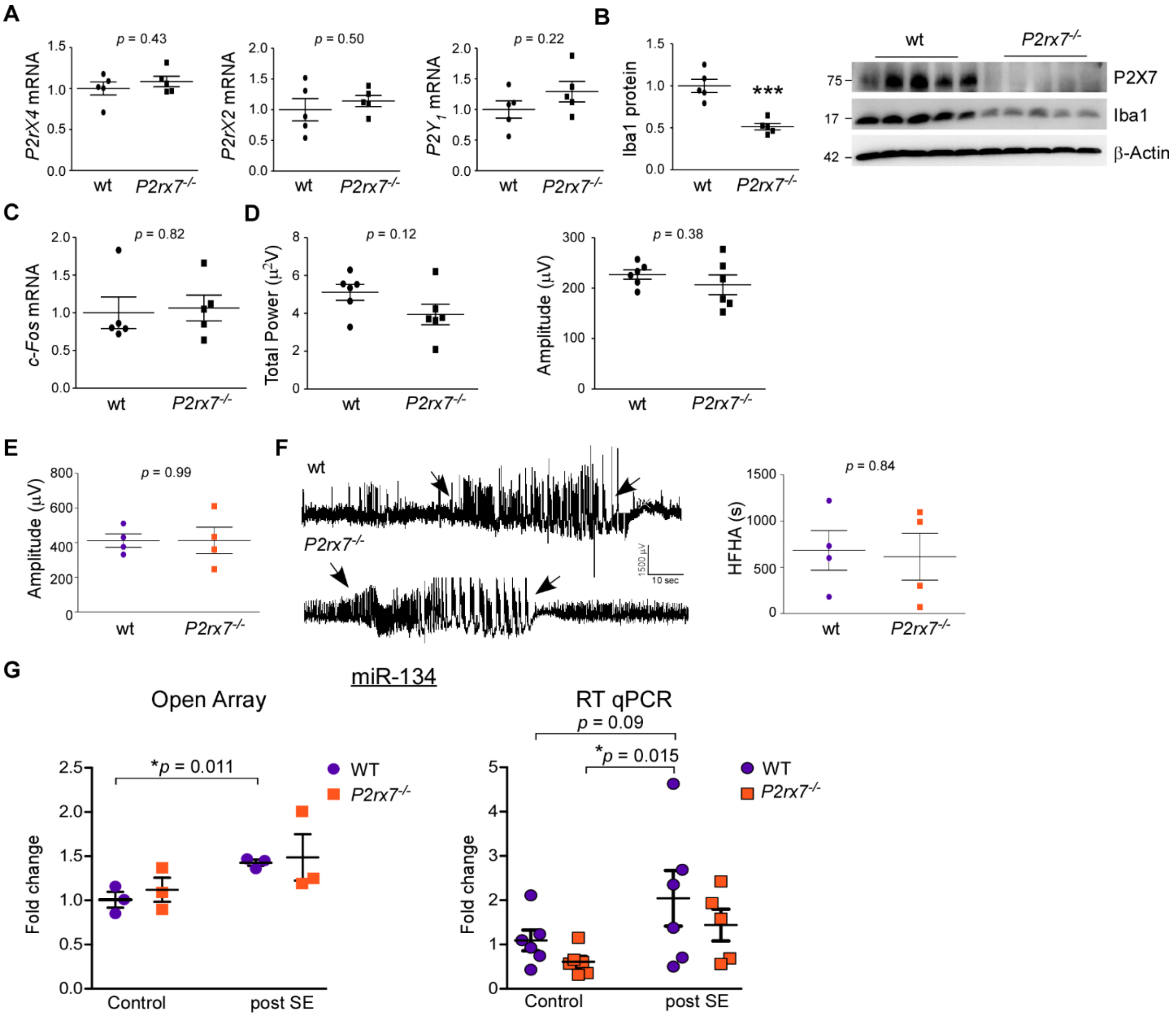

(A) Graphs showing similar *P2rx2*, *P2rx4* and *P2ry1* transcript levels in the hippocampus between naïve wt and *P2rx7*$^{-/-}$ mice (n = 5 per group). (**B**) Less Iba1 protein levels in the hippocampus of naïve *P2rx7*$^{-/-}$ mice when compared to wt mice (n = 5 per group). (**C**) Similar *c-Fos* transcript levels in the hippocampus of naïve wt and *P2rx7*$^{-/-}$ mice (n = 5 per group). (**D**) Similar baseline

EEG recordings between wt and *P2rx7*$^{-/-}$ mice (n = 6 per group). (**E**) Graph showing no difference in the EEG amplitude between wt and *P2rx7*$^{-/-}$ mice during 40 min of status epilepticus (n = 4 per group). **(F)** Representative EEG traces showing examples of high frequency and high amplitude (HFHA) spiking taken during status epilepticus (see arrows) and corresponding graph showing no difference in the HFHA seizure burden between wild-type and *P2rx7*$^{-/-}$ mice (n = 4 per group). **(G)** Graph showing increased miR-134 levels in the hippocampus of wild-type mice and *P2rx7*$^{-/-}$ mice post-status epilepticus (post-SE) as detected via OpenArray (n = 3 per group) and via individual qPCR (n = 6 for wt (Control and post-SE) and *P2rx7*$^{-/-}$ (Control) and n = 5 for *P2rx7*$^{-/-}$ post-SE).

Supplementary Figure S2. *MicroRNAs previously reported to undergo seizure-induced expression changes validated via OpenArray.*

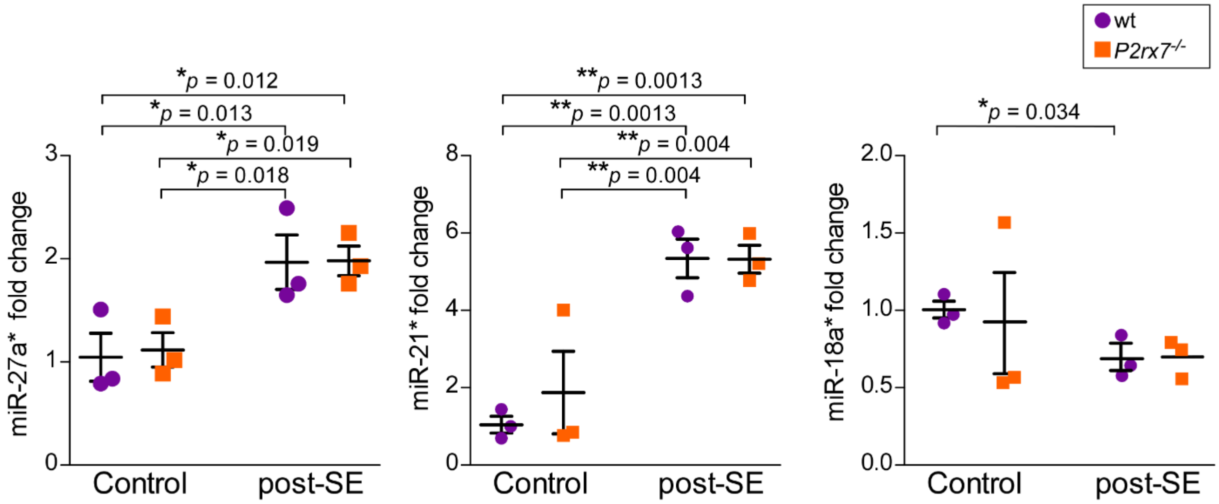

Graphs showing expression of miR-27a*, miR-21* and miR-18a* in different conditions as detected via the OpenArray (n = 3 per group). Note, all three miRNAs undergo similar expression changes post-status epilepticus (post-SE) in wt and *P2rx7*$^{-/-}$ mice. ANOVA with Fisher's correction. *p < 0.05, **p < 0.01

Supplementary Figure S3. *Volcano plot representation of differentially expressed miRNAs.*

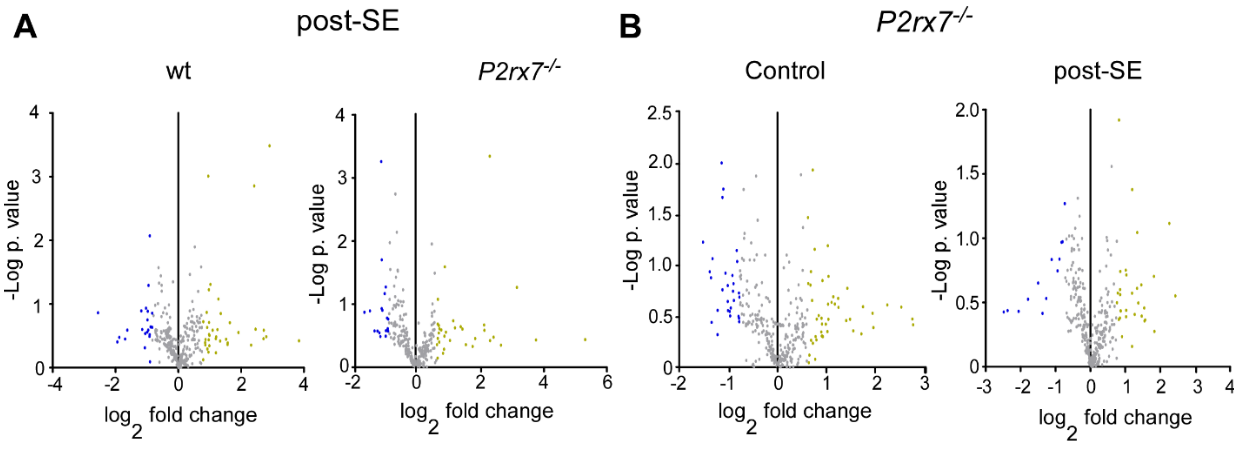

(A) Differentially expressed miRNAs post-status epilepticus in wild-type (wt) and *P2rx7*$^{-/-}$ mice normalized to wt controls. In yellow the fold changes of the up-regulated miRNAs and in blue the fold changes of down-regulated miRNAs. **(B)** Differentially expressed miRNAs in *P2rx7*$^{-/-}$ mice, in control condition normalized to wt control and post-status epilepticus normalized to wt post-status epilepticus. In yellow the fold changes of up-regulated miRNAs and in blue fold changes of down-regulated miRNAs.

Supplementary Figure S4. *Network organization of predicted miRNA target genes during control conditions.*

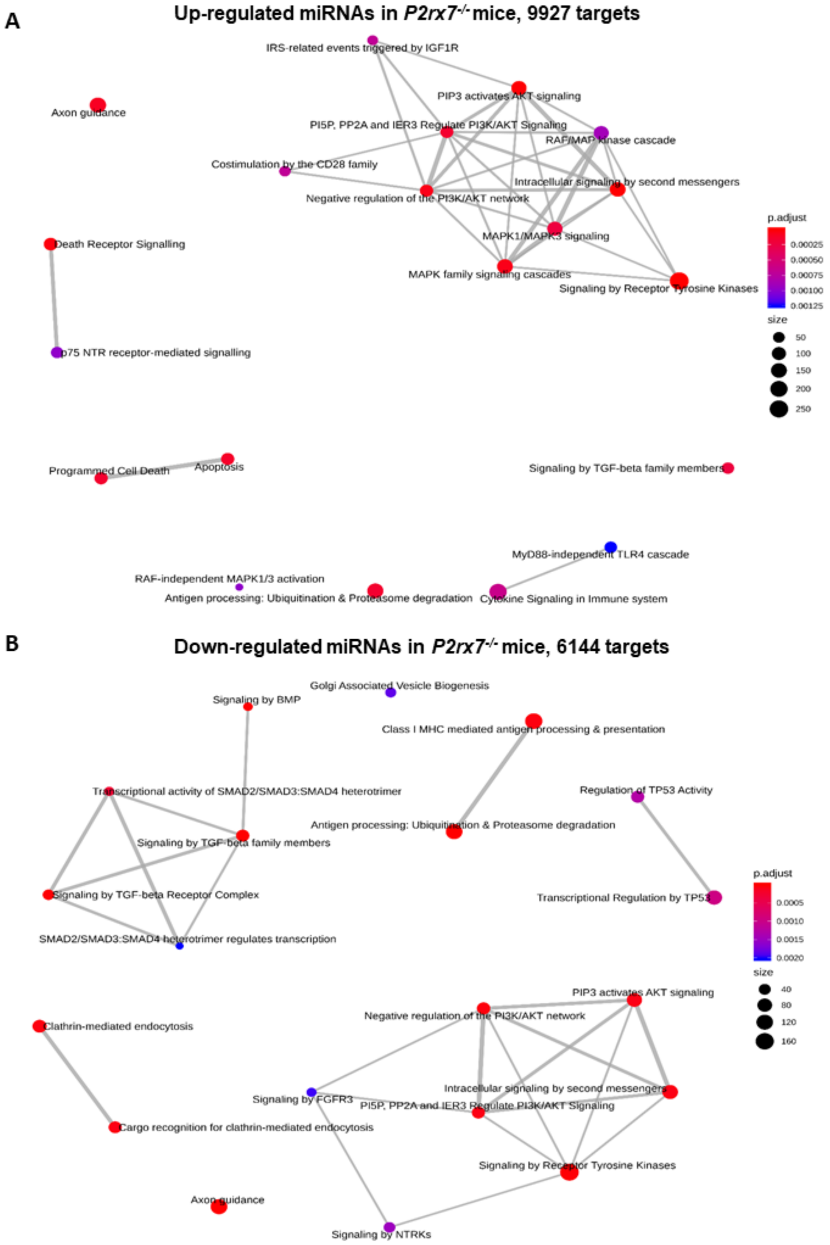

Network map of GO terms significantly enriched among target genes targeted via **(A)** up-regulated miRNAs and **(B)** down-regulated miRNAs in *P2rx7*$^{-/-}$ mice during control condition**.**

Supplementary Figure S5. *Network organization of predicted miRNA target genes post-status epilepticus.*

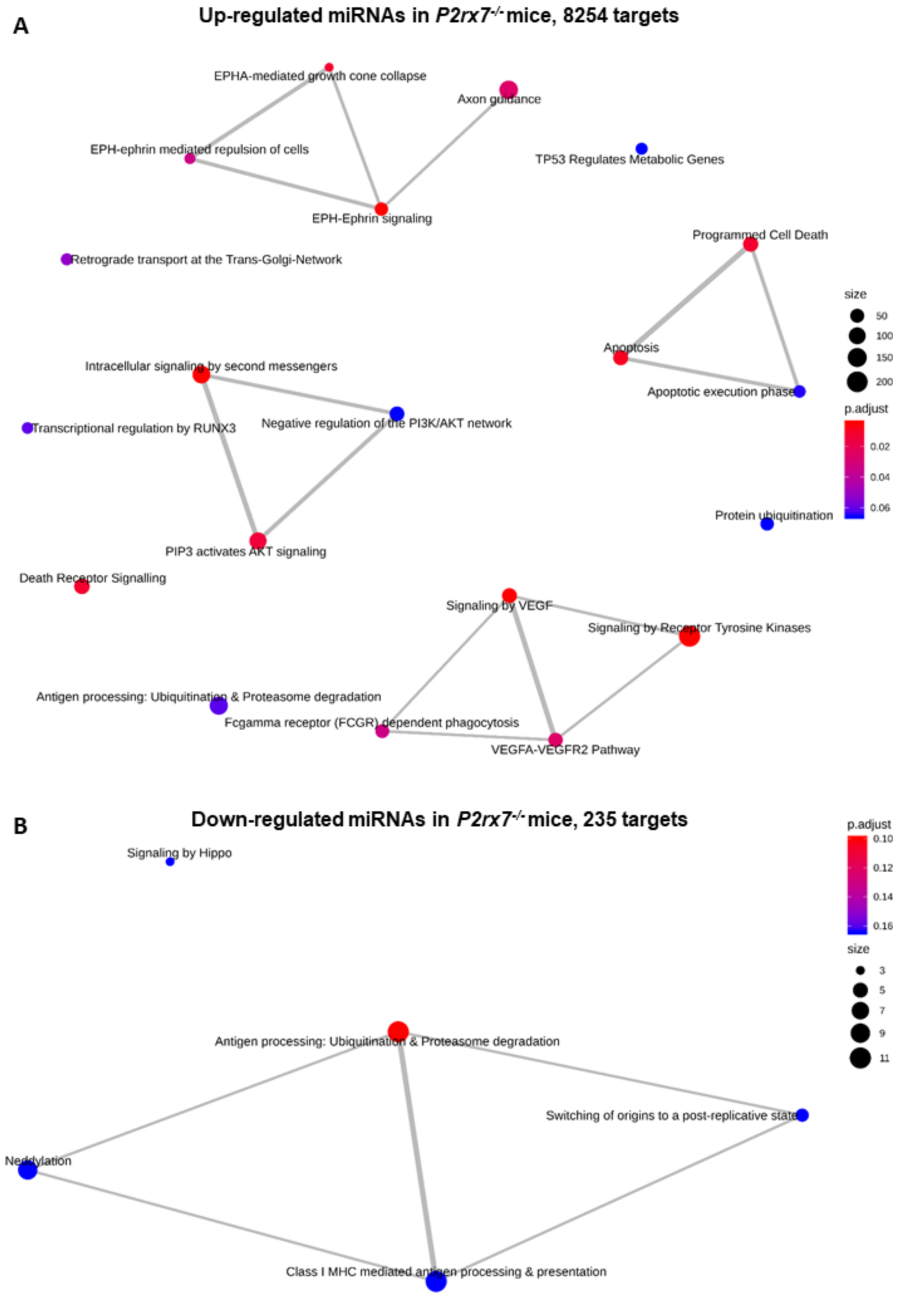

Network map of GO terms significantly enriched among genes targeted via **(A)** up-regulated and **(B)** down-regulated miRNAs in *P2rx7*$^{-/-}$ mice post status epilepticus**.**
